# Dynamic recruitment of microRNAs to their mRNA targets in the regenerating liver

**DOI:** 10.1186/1471-2164-14-264

**Published:** 2013-04-18

**Authors:** Jonathan Schug, Lindsay B McKenna, Gabriel Walton, Nicholas Hand, Sarmistha Mukherjee, Kow Essuman, Zhongjie Shi, Yan Gao, Karen Markley, Momo Nakagawa, Vasumathi Kameswaran, Anastassios Vourekas, Joshua R Friedman, Klaus H Kaestner, Linda E Greenbaum

**Affiliations:** 1 Department of Genetics and Institute for Diabetes, Obesity and Metabolism, Perelman School of Medicine, University of Pennsylvania, Philadelphia, Pennsylvania, USA; 2 Department of Pediatrics, The Children’s Hospital, University of Pennsylvania, Philadelphia, Pennsylvania, USA; 3 Departments of Cancer Biology and Medicine, Thomas Jefferson University, 519 BLSB, 233 S. 10th Street, Philadelphia, PA, 19107, USA; 4 Department of Cancer Biology, University of Pennsylvania, Philadelphia, Pennsylvania, USA; 5 Department of Pathology and Laboratory Medicine, University of Pennsylvania, Philadelphia, Pennsylvania, USA

**Keywords:** Liver, Hepatectomy, HITS-CLIP, ceRNA, microRNA, Cell cycle

## Abstract

**Background:**

Validation of physiologic miRNA targets has been met with significant challenges. We employed HITS-CLIP to identify which miRNAs participate in liver regeneration, and to identify their target mRNAs.

**Results:**

miRNA recruitment to the RISC is highly dynamic, changing more than five-fold for several miRNAs. miRNA recruitment to the RISC did not correlate with changes in overall miRNA expression for these dynamically recruited miRNAs, emphasizing the necessity to determine miRNA recruitment to the RISC in order to fully assess the impact of miRNA regulation. We incorporated RNA-seq quantification of total mRNA to identify expression-weighted Ago footprints, and developed a microRNA regulatory element (MRE) prediction algorithm that represents a greater than 20-fold refinement over computational methods alone. These high confidence MREs were used to generate candidate ‘competing endogenous RNA’ (ceRNA) networks.

**Conclusion:**

HITS-CLIP analysis provide novel insights into global miRNA:mRNA relationships in the regenerating liver.

## Background

microRNAs (miRNAs), 22–23 nucleotide noncoding RNAs, contribute to the control of diverse developmental, growth, and disease processes [1,2]. Abnormal expression of miRNAs has been established in cancers, with miRNAs functioning as either tumor suppressors or oncogenes. MicroRNAs decrease expression of mRNA targets by either destabilization of mRNA or inhibition of protein translation [3]. MicroRNAs are thought to target mRNAs through binding of nucleotides at position 2–8 of the miRNAs (the so-called ‘seed region’) to a complementary sequence in the mRNA [4,5].

While differential expression of miRNAs has been determined in multiple contexts, the validation of physiologic miRNA targets has proven to be difficult. Modulation of miRNA levels using gain- and loss-of-function approaches have identified some mRNA targets; however, concerns exist regarding the potential for indirect and off-target effects, particularly in the case where a miRNA is overexpressed. Currently available miRNA target prediction algorithms produce large numbers of potential targets; however, very few of these targets have been experimentally validated. Many current algorithms mine sequences limited to the 3^′^UTR of mRNAs, and therefore do not identify miRNAs that target the coding region and 5^′^UTR, even though miRNA targeting to all mRNA regions has now been experimentally validated [5-11]. Furthermore, the ability of miRNAs to target mRNAs with only partial complementarity [12] indicates that in many cases identification of miRNA targets may not be possible based on seed match complementarity alone [10]. Consequently, better methods are needed to identify bona fide miRNA targets.

Recently, Pandolfi and colleagues have proposed an ‘mRNA code’, in which competing endogenous mRNAs (ceRNAs) communicate with each other through crosstalk between shared microRNA regulatory elements (MRE) [10,13]. An important prediction of this model is that competition for a common pool of miRNAs could result in de-repression of one mRNA following robust activation of a second mRNA containing the same MREs. Therefore, identification of MREs and mRNA:miRNA targeting relationships is an essential step towards understanding the role of miRNAs in the regulation of complex biological responses.

The partial hepatectomy model in rodents has been used extensively to investigate the mechanisms responsible for hepatic growth and proliferation, and is the best *in vivo* model of synchronous cell cycle progression in mammals [14,15]. Following surgical removal of two thirds of the liver, hepatocytes and nonparenchymal cells rapidly reenter the cell cycle, replicate, and restore the original mass of the liver within 10–14 days [14]. This process involves a complex regulatory cascade of cytokine signals and transcriptional regulators that coordinate cell cycle progression while maintaining homeostasis [15,16]. An essential contribution of miRNAs in this regenerative response has been supported by a recent study in which mice with genetic deletion of the DROSHA cofactor DGCR8, a factor required for microRNA biogenesis, exhibited markedly impaired hepatocyte proliferation after partial hepatectomy [17]. Although changes in expression of miRNAs after partial hepatectomy and in liver-graft models have been reported using array-based assays [17-24], only a small number of targets have been validated [22,23,25,26]. Importantly, the identification of the subset of mRNAs that are regulated by miRNAs in the regenerating liver is far from complete, in part due to the large number of possible mRNA:miRNA targeting relationships predicted by computational approaches.

We hypothesized that recruitment of miRNAs to the RISC, or RNA-induced silencing complex, which contains the partially base-paired miRNA and its mRNA target, would be a more informative method to assess miRNA activity during liver regeneration, since changes in the overall expression level of miRNAs reported thus far do not take into consideration changes in target mRNA expression or MRE accessibility. To identify which miRNAs participate in the regenerative process and to identify their targets, we used a technique pioneered by the Darnell laboratory in which UV cross-linked miRNA:mRNA complexes are immunoprecipitated with an antibody to Argonaute, an essential component of the RISC, and then subjected to deep sequencing analysis [27-29]. We found that a subset of miRNAs is dynamically recruited to the RISC during liver regeneration, and that for the majority of these miRNAs, RISC recruitment did not correlate with changes in overall expression. Furthermore, we exploited the comparison of RNA-seq and RISC recruitment data to identify those mRNAs whose recruitment to the RISC was highly enriched relative to their overall abundance, and which are therefore likely to be regulated by miRNAs.

## Results and discussion

Changes in expression of miRNAs, mRNAs and/or accessibility of microRNA regulatory elements (MREs) to their complementary miRNAs are likely to affect miRNA recruitment to the RISC [4]. We hypothesized that miRNA activity would be more accurately determined by quantification of miRNA abundance in the RISC than by changes in miRNA levels alone. To this end, we determined miRNA recruitment at selected time points posthepatectomy previously shown to correspond to the G_1_ (1h), S (36H) and M (48H) phases of the hepatocyte cell cycle, and in the quiescent liver, corresponding to G_0_[30,31]. Cell cycle stage was confirmed by activation of genes associated with G_1_ (jun, fos, myc), S (cyclin D1) and M (Foxm1) cell cycle phases determined by RNA-seq analysis of samples from these time points (Additional file 1: Figure S1). Following UV-crosslinking of the RISC to microRNA and mRNA, we quantified miRNAs immunoprecipitated with an antibody that recognizes Argonaute 1 through 4 proteins and therefore immunoprecipitates all RISCs [32] by ultra-high throughput sequencing. We calculated the loading microRNA relative to all others for each replicate as reads per million (RPM). We then employed a stringent cut-off of 100 RPM through which we detected 226 miRNAs in the RISC complex at one or more time points examined (Additional file 2: Table S1 contains loading data for the 632 miRNAs that had any aligning reads). An important issue for any comparison across time is global normalization of the data. Because the average levels of RISC-associated microRNAs were relatively unchanged over time, we employed additional global normalization, as well as a stricter 1,000 RPM cut-off, to identify differentially-loaded microRNAs.

**Figure 1:**
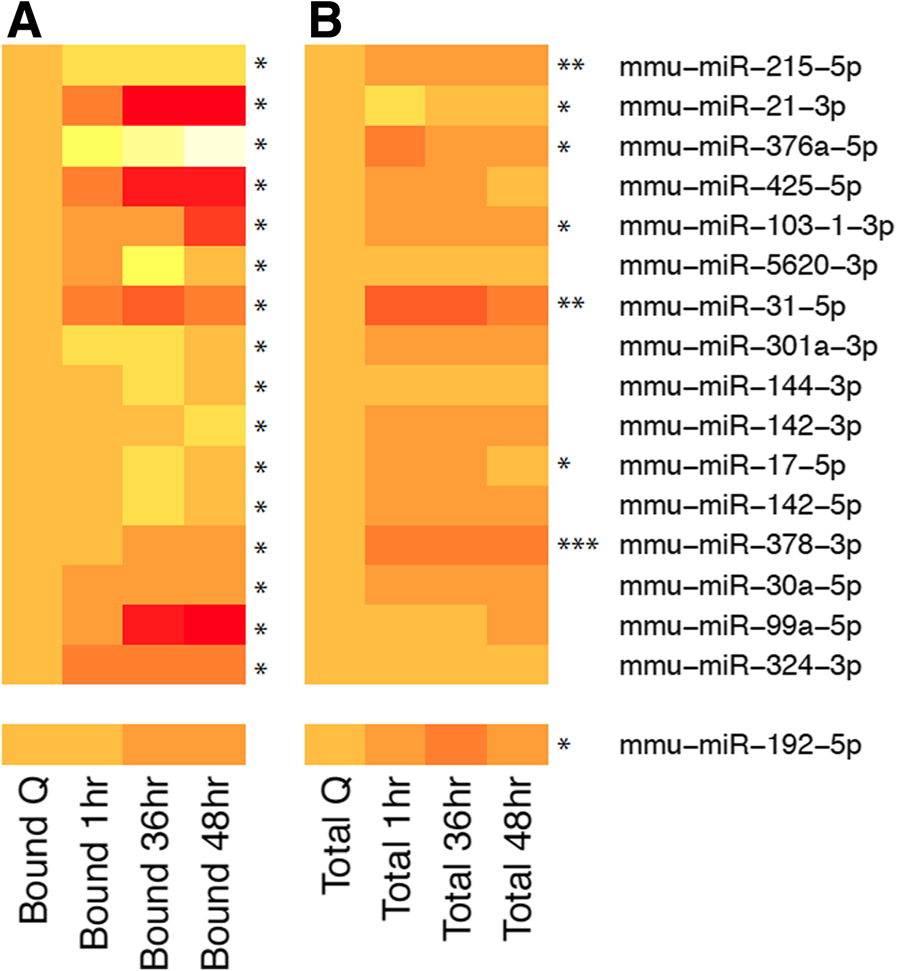
**Changes in miRNA total regulatory load on mRNAs and total expression at indicated time points posthepatectomy compared to quiescent liver.** (**A**) Sixteen miRNAs were found to have altered levels of RISC-loading during the time course of the experiment. (FDR < = 20%). miR-192-5p has the same seed sequence as mir-215-5p so was included for comparison. (**B**) The total expression of 16 of these miRNAs was measured using taqMan qRT-PCR at indicated times after partial hepatectomy relative to quiescent liver. N = 2 samples. Significance was calculated by ANOVA. Levels are normalized to quiescent liver for display purposes. Red is lower, yellow-white is higher. A taqMan probe was not available for miR-5620 and was therefore not analyzed.

Using these criteria, 16 miRNAs exhibited a significant increase (9) or decrease (7) in RISC recruitment at one or more time points posthepatectomy relative to the quiescent liver, with a dynamic range exceeding five-fold in an interval as short as one hour (Figure 1A). We did not detect differences in RISC recruitment of mir-21-5p, a microRNA that had been shown previously to be significantly induced 18 hours posthepatectomy [17]. This may reflect a lack of correspondence between overall expression of mir-21-5p and RISC recruitment. It is also conceivable that mir-21-5p RISC enrichment is increased selectively at 18 hours posthepatectomy, a time point that was not examined in the current study. Our HITS-CLIP analysis did, however, predict several previously confirmed mir-21-5p targets in the regenerating liver including the anti-apoptotic factor, Btg2 [17] and the NF-κB inhibitor Pellino 1 [18].

The change in recruitment was extremely rapid for the majority of these miRNAs. For example, RISC recruitment of mir-215 increased approximately 5.6-fold by 1 h posthepatectomy, and was also similarly enriched at the time point corresponding to hepatocyte S phase (36 h) relative to quiescent liver. Surprisingly, mir-215 and mir-192, which contain the same seed sequence (Additional file 3: Figure S2), are both regulated by p53 [33,34] and expressed at similar levels after hepatectomy (Figure 1B), followed different patterns of RISC recruitment. This discordance could reflect requirements for sequences outside of the seed region of the two miRNAs for binding to their targets or interaction with RNA binding proteins.

This rapid change in miRNA recruitment to the RISC is unlikely to be due solely to an increase in miRNA transcription, and likely reflects enhanced miRNA processing, stability, changes in miRNA subcellular localization, and/or changes in the abundance of specific mRNA targets or RNA binding proteins. Several miRNAs exhibited changes in RISC recruitment at later time points posthepatectomy. For example, significant RISC enrichment of mir-142-3p occurred 48 h posthepatectomy. This miRNA has been shown recently to inhibit RAC1-mediated colony formation migration and invasion in HCC cell lines [35] and could have a similar function in proliferating hepatocytes. We investigated whether changes in miRNA recruitment to the RISC were associated with corresponding changes in overall miRNA abundance during the regenerating time course (Figure 1A, B). Importantly, with the exception of mir-31, mir-144 and mir-378, we found no correlation between miRNA expression and RISC recruitment and for many of the miRNAs examined, changes in RISC recruitment and overall expression were inversely correlated. Overall, the dramatic changes in the levels of actively engaged miRNAs over a short time frame emphasize the necessity to determine miRNA recruitment to the RISC rather than relying on simple determination of miRNA abundance in order to fully assess the impact of miRNA regulation.

**Figure 2:**
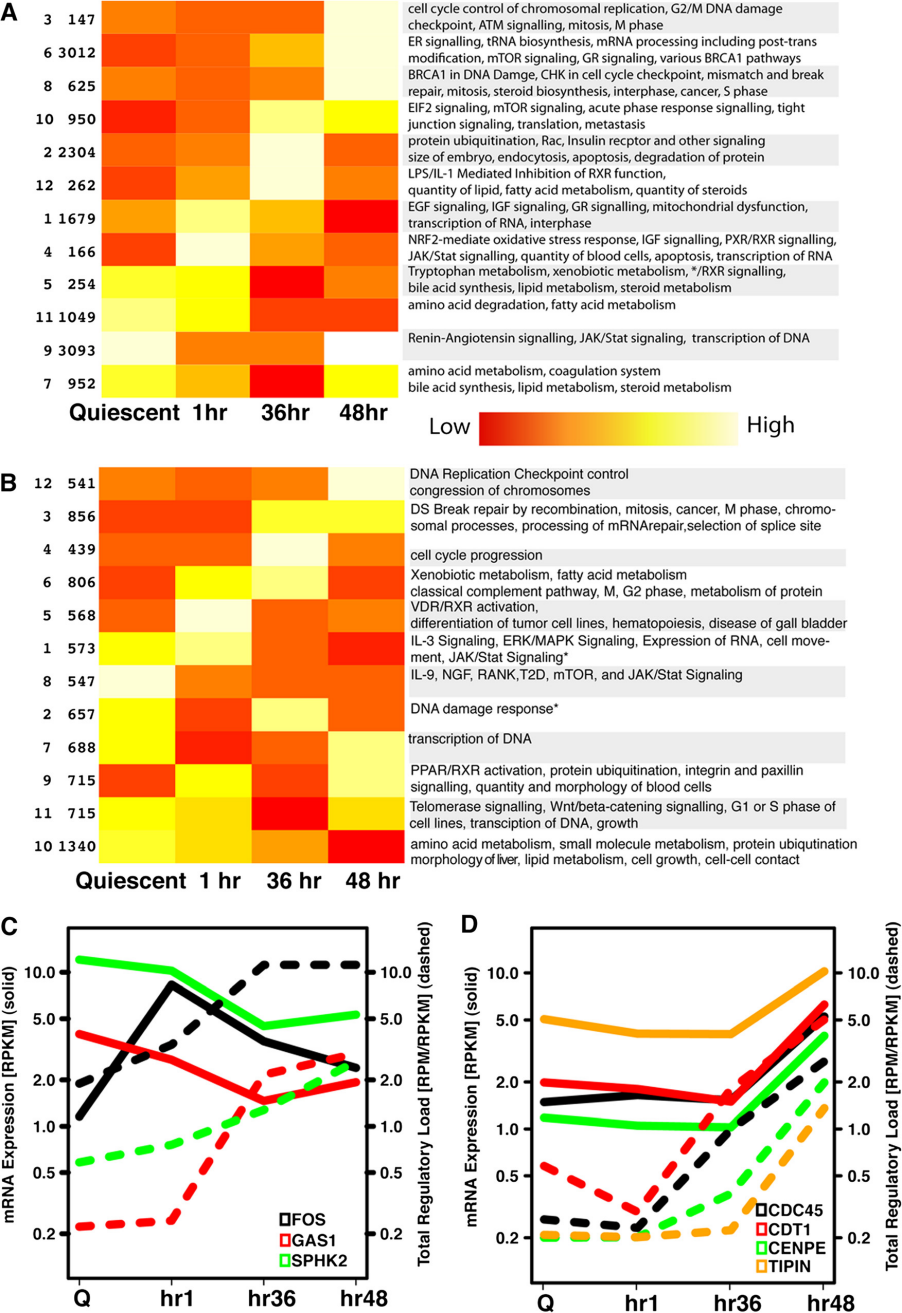
**Clustering and functional analysis of mRNA and total regulatory load profiles.** k-means clustering was used to summarize the main patterns of mRNA levels (**A**) and total regulatory load (**B**) across the regeneration profile. Within each cluster we identified enriched pathways and functions. The numbers on the left of each cluster indicate the cluster number (referenced in Additional files 5 and 6) and the number of genes in the cluster. Contrasting the mRNA expression levels with the total regulatory load (TRL) for a set of genes in an enriched pathway reveals information about the potential effect of miRNA regulation. Genes in (**C**) and (**D**) display different regulatory relationships. Panel C contains genes for which increased TRL precedes or coincides with decreased overall expression of immediately early (Fos) or antiproliferative target genes (GAS1 and SPHK2), whereas in Panel D, containing genes involved in the DNA replication checkpoint pathway, the TRL (dashed lines) begins to increase at 36 hours posthepatectomy (S phase peak), 12 hours prior to the increase of the relevant mRNAs (solid lines).

Next, we turned our attention to the mRNAs that were identified in the RISC using our HITS-CLIP assay. We speculated that miRNA regulation would be more significant for those mRNAs that were highly enriched in the RISC relative to their overall abundance in the tissue. Therefore, to identify ‘expression-weighted footprints’, we used RNA-seq to quantify total mRNA expression in quiescent liver, and 1 h, 36 h and 48 h posthepatectomy. Next, we calculated RISC enrichment for all mRNAs relative to their overall abundance (Additional file 4: Table S2). Using the enrichment-weighted values for all mRNAs, we applied k-means cluster analysis to identify groups of genes that exhibited similar changes in RISC recruitment across the posthepatectomy time course. Pathway analysis applied to these gene clusters identified specific networks and biological functional categories that were highly significant (Figure 2A, B and Additional file 5: Table S3 and Additional file 6: Table S4). The overall mRNA expression levels follow the expected expression patterns, with genes involved in basic liver function pathways enriched in the quiescent liver (e.g. clusters 5, 11), and cell cycle genes increasing at 48 hours posthepatectomy (e.g. clusters 3, 8). Cell cycle progression and checkpoint control gene clusters were maximally enriched in the RISC (B) at 36 h (cluster 3: mitosis p < 1.92E-10) and 48 h (cluster 12, DNA replication checkpoint, p < 2.71E-07). In contrast to the pattern of cell cycle gene enrichment in the RISC, genes involved in amino acid metabolism (p < 2.79E-07), lipid metabolism (Cluster 10, synthesis of lipids, p < 3.41E-07), and cell growth (Cluster 10, p <6.45E-08) were gradually decreased in the RISC following hepatectomy. Growth factors expressed in hepatocytes during regeneration including Fgf1 and VegfA were enriched the RISC (Additional file 4: Table S2) suggesting that these factors are also regulated by miRNAs. HGF, which is synthesized by hepatocyte stellate and endothelial cells, was also highly enriched in the RISC in quiescent liver followed by a rapid and sustained fall in enrichment that did not normalize until 48 h after partial hepatectomy (Additional file 4: Table S2). Mobilization of preexisting HGF from extracellular matrix by urokinase plasminogen activator provides a rapidly available source of this growth factor that is required to stimulate hepatocyte proliferation [36]. Our findings suggest that relief of miRNA inhibition of HGF may serve to further enhance HGF mRNA levels during the regenerative response.

Regulation of mRNAs by miRNAs may either “fine-tune” or significantly inhibit target mRNA expression [4]. To investigate the potential regulatory function of miRNAs for their targets in the regenerating liver, we plotted total gene expression and RISC recruitment for two clusters of genes known to be important in the regenerative process after partial hepatectomy [37]. The total regulatory load (TRL) of Gas1, an inhibitor of proliferation, increases coincident with a decrease in its overall expression, suggesting that its inhibition by miRNAs may facilitate the G_1_/S transition [38] (Figure 2C). SPHK2 represses transcriptional activation of the immediate-early growth gene Fos via inhibition of histone acetylation [39]. These findings suggest that Fos and SPHK2 inhibition by miRNAs could cooperatively contribute to the resolution of Fos activation posthepatectomy. In contrast, the TRL of mRNAs encoding proteins involved in DNA checkpoint regulation increases at 36 hours posthepatectomy, prior to the observed overall increase in mRNA at 48 hours posthepatectomy (Figure 2D). This temporal relationship suggests that miRNAs may limit and/or delay the increase in expression of replication checkpoint genes during hepatocyte S phase, allowing for completion of DNA replication.

**Figure 3:**
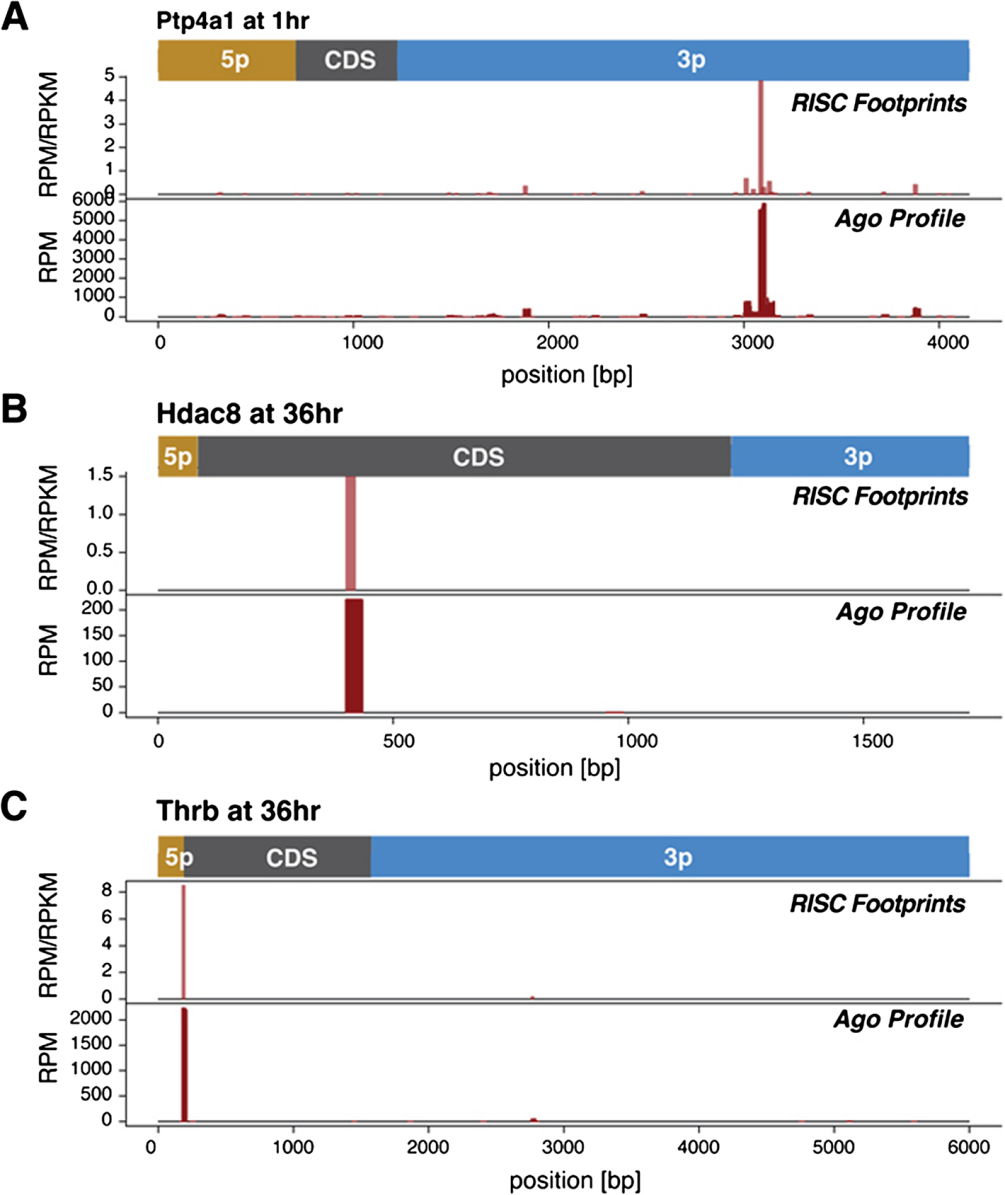
**Examples of Ago footprint location and discovery.** The profiles of coverage by HITS-CLIP sequence reads for three genes (**A**) Ptp4a1 (PRL-1) (**B**) HDAC8 and (**C**) TRB (thyroid receptor beta); indicated time points are shown.

The majority of miRNA target prediction algorithms utilize complementary seed match pairing to the 3^′^UTR of candidate mRNAs, although recent reports have identified miRNA regulatory sequences in coding region (CDS) and 5’UTR regions [5-11]. Most of the Ago footprints aligned to sequences in the 3^′^UTR of mRNAs, but we also found footprints in the CDS and 5’UTR in a subset of mRNA targets (Figure 3 and Additional file 7: Table S5). Footprints were identified for many genes with known roles in liver growth. For example, we identified a dominant footprint in the 3^′^UTR of Ptp4a1 (PRL-1), an immediate-early gene in the regenerating liver associated with cell growth (Figure 3A) [40]. A unique footprint was found in the coding region of the histone deacetylase, HDAC8, a modulator of estrogen related receptor alpha activity (Figure 3B) [41]. An example of a strong 5^′^UTR footprint is shown for thyroid receptor beta (THRB), a regulator of hepatic lipogenesis (Figure 3C) [41,42].

**Figure 4:**
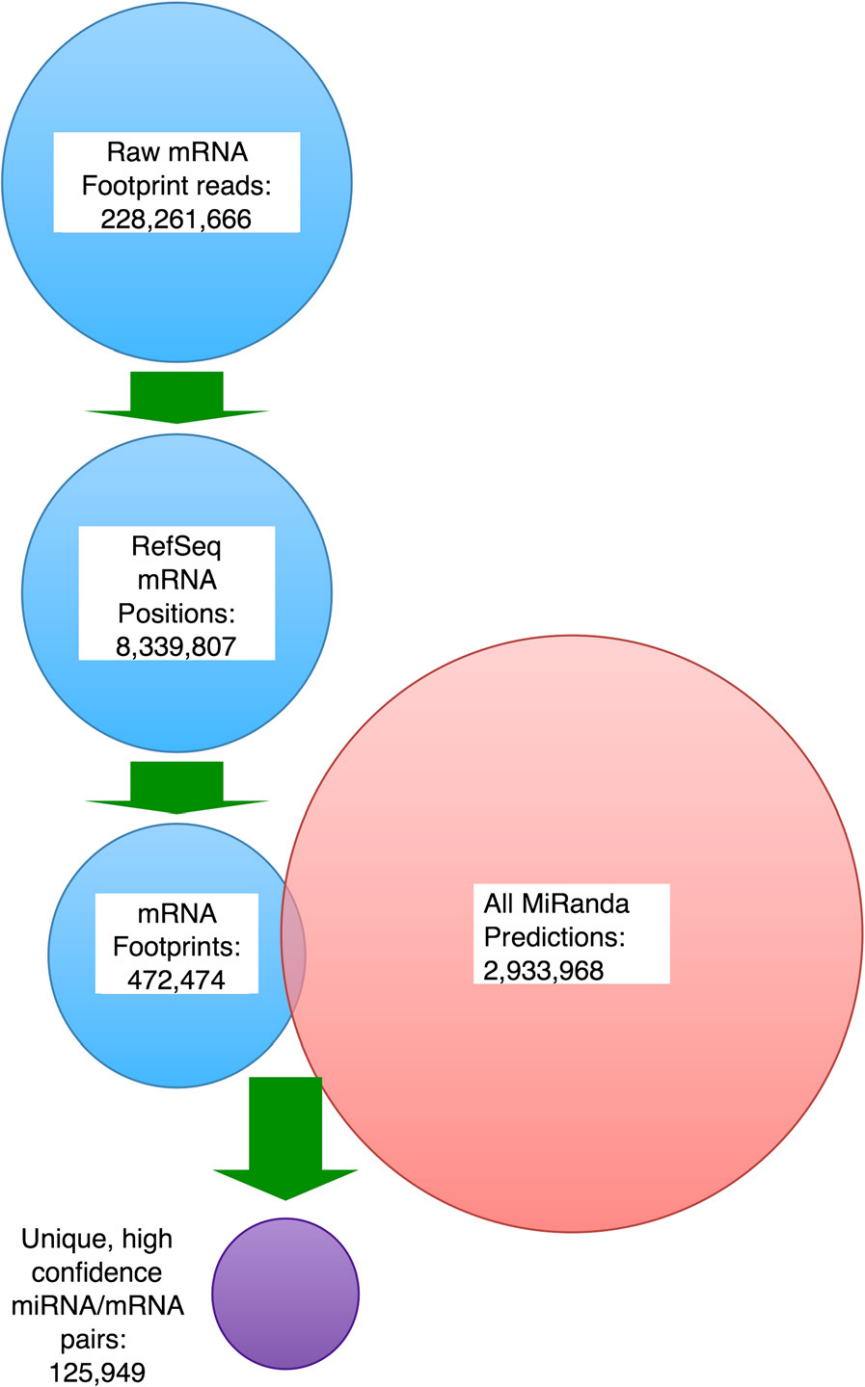
**Method for high confidence prediction of miRNA–mRNA targeting relationships.** HITS-CLIP reads for mRNA fragments from all our time-points were pooled for the purpose of predicting miRNA/mRNA targeting relationships in the liver. Over 228 million raw sequence reads were aligned to RefSeq mRNAs to yield over 8 million fragment start positions. These mRNA fragments were coalesced into ‘footprints’ anchored by the locally strongest accumulation of reads, yielding 472,474 mRNA footprints. These footprints represent the mRNAs that are targeted to the argonaute-containing RISC at any time during liver regeneration. Next, these mRNA footprints were intersected with all possible predictions of miRNA/mRNA targeting relationships obtained from miRanda [43], yielding 125,949 unique and high confidence miRNA/mRNA pairs. Note that the combination of the computational approach (miRanda) with the experimental approach (HITS-CLIP) refined the computational predictions by more than 20-fold. Circles not drawn to scale.

The mRNA fragments identified by HITS-CLIP are predicted to contain or be adjacent to miRNA regulatory elements (MREs), and therefore should enable us to refine the computational predictions of miRNA-mRNA pairs by reducing the ‘search space’ in which to evaluate matches to miRNA seed sequences. For this purpose, we began by compiling all mRNA fragments we obtained from the Argonaute immunoprecipitation, and aligned them to all RefSeq mRNAs. Next, we catalogued all starting positions of these mRNA fragments. These mRNA fragments were then coalesced into ‘footprints’ by the locally strongest accumulation of reads, yielding 472,474 footprints, or sites of RISC occupancy in mRNAs. Finally, these footprints were intersected with all predicted mRNA:miRNA targeting relationships obtained by miRanda, when applied to all known miRNAs and RefSeq mRNAs. This refinement of the computational approach using our experimental data on RISC footprints resulted in 125,949 unique and high confidence miRNA/mRNA pairs, which is a greater than 20-fold refinement of the results obtained from computational target prediction alone (Figure 4).

To assess the accuracy of our predicted miRNA/mRNA pairs, we compared our results to a set of genes whose expression was altered in liver in response to miR-29a-3p antisense oligonucleotide treatment [44]. Hand and colleagues identified 78 genes that were up-regulated upon blocking miR-29a-3p, and an additional 65 genes that were down-regulated. We identified 381 predicted targets of miR-29a-3p in quiescent liver using the methods summarized in Figure 4. Of these predicted targets, 10 (p = 4.16e-06; Fisher exact test, followed by Benjamini-Hochberg correction for multiple testing) were found in the up-regulated set in Hand and colleagues [44] whereas only 3 (p = 0.228) were found in the down-regulated set. Performing the same analysis for all miRNAs, we found the targets of miR-29a-3p to have the second most significant overlap with the up-regulated set obtained experimentally [44]. The most significant overlap was with miR-328-3p that had a similar overlap (10 genes in common with 282 targets and 4 targets in common with miR-29a-3p). Next, we performed a similar comparison of the miR-122-5p targets identified in the quiescent liver [45,46], and obtained a similar trend. We identified 748 target genes and 866 target mRNAs for miR-122-5p. The overlap between our targets and the microarray data were significant for the genes up-regulated upon interference with miR-122-5p activity (36 of 206 transcripts p = 3.87e-09 for Esau and 42 of 363 p = 3.69e-15 for Krutzfeldt), and not significant for the down-regulated genes (32 of 619 p = 1 for Esau and 16 of 305 p = 1 for Krutzfeldt). For both sets of up-regulated genes, miR-122-5p was the miRNA with the most significant overlap with the microarray data. Based on the differences between the experimental models, our results are likely to underestimate the true extent of mir-29a-3p or miR-122-5p target overlap. Nevertheless, these findings provide strong support for the validity of our target prediction approach.

**Figure 5:**
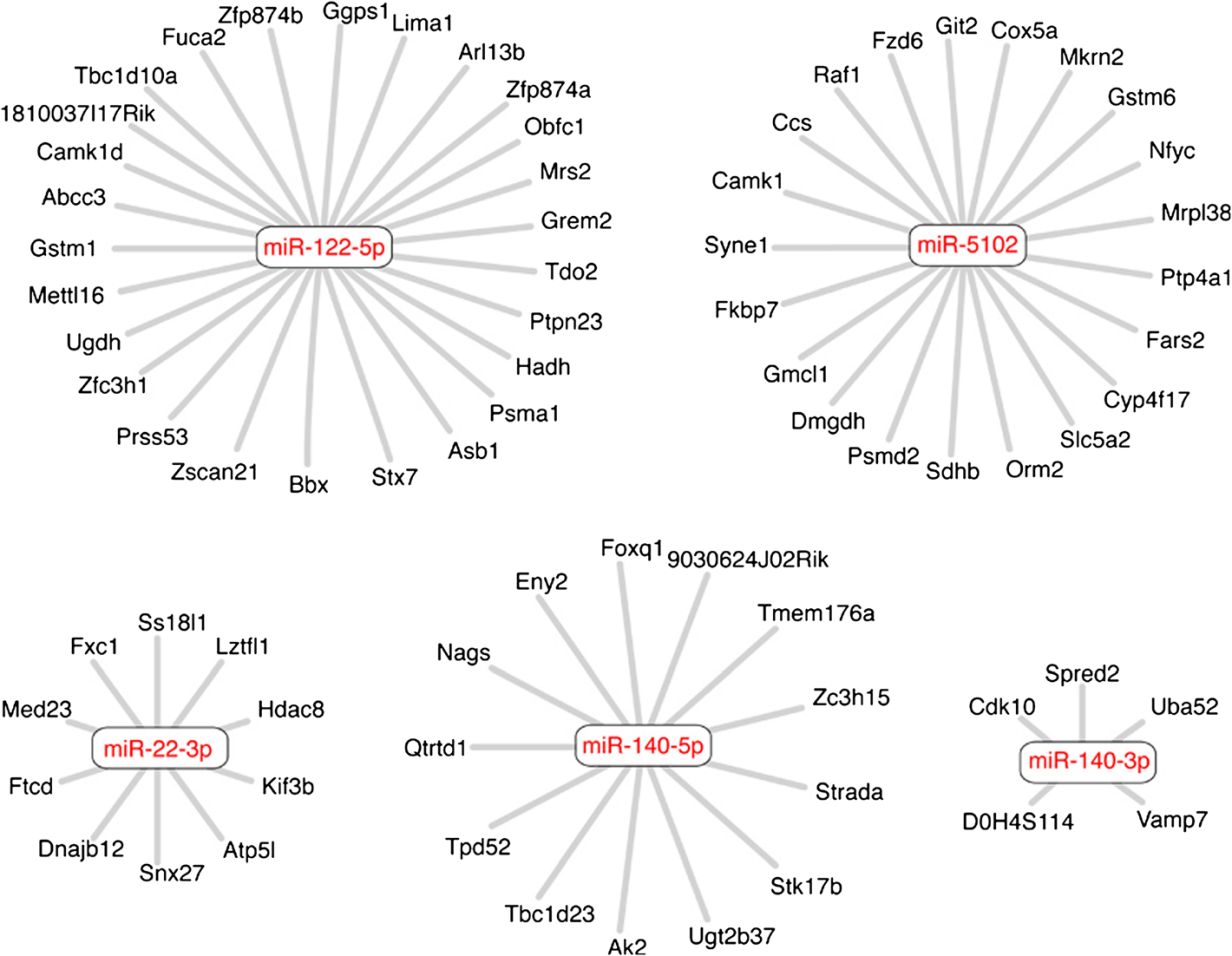
**CeRNA Networks. In a competing endogenous RNA (ceRNA) network, RNAs regulated by a single miRNA can compete with other mRNAs for that miRNA and control its effect on other targets.** We identified mRNAs that had a single strong RISC footprint that contained a single highly-loaded miRNA and built ceRNA networks. Networks for miR-122-5p, miR-5102, mir-22-3p, mir-140-5p and mir-140-3p are shown.

A prediction of the ceRNA hypothesis is that mRNAs containing the same MREs will compete for a common pool of miRNAs [13]. We postulate that activation of a subset of growth regulatory mRNAs in the regenerating liver may be due in part to relief of inhibitory effects of other highly induced mRNAs that compete for the same pool of miRNAs. At present the extent of these ceRNA networks has been difficult to establish, due to the aforementioned limitations of the computational target prediction algorithms. Therefore, we applied our mRNA:miRNA targeting relationship predictions to build candidate ceRNA networks in the regenerating liver, selecting those mRNAs that had a single, strong miRNA footprint. Several examples of predicted miRNA ceRNA networks identified by our analysis are shown in Figure 5. Two members of the mir-140-3p ceRNA network, Spred2 and Vamp7, exert opposite effects on MAPK signaling pathways via inhibition of Raf phosphorylation [47] and endocytosis of the EGF receptor, respectively [48]. Thus, competition by these two mRNAs for mir-140-3p could balance positive and negative regulation of MAPK signaling in the regenerating liver, providing an example for the potential functional relevance of ceRNA networks.

## Conclusions

A significant challenge in the field of microRNA biology is to understand physiologic miRNA:mRNA regulatory relationships in highly complex *in vivo* systems. We have demonstrated here that the HITS-CLIP assay can be applied to a complex model of growth and proliferation. We demonstrate that dynamic changes in miRNA recruitment to the RISC in most cases did not correlate with overall expression patterns in the regenerating liver, indicating that assessment of expression levels alone does not reflect miRNA activity. This lack of correspondence between RISC recruitment and miRNA expression is likely to be influenced by multiple factors including contributions of RNA binding proteins [49] ratio of target mRNA:targeting miRNA, flanking sequence homology [50] and changes in miRNA subcellular localization [51]. Using miRNA sensory and decoy libraries, Mullokandov and colleagues recently showed that only highly abundant miRNAs showed significant target repression, and in some instances were also less active based on a high mRNA:miRNA ratio or nuclear localization [51]. These findings suggest that the small changes in miRNA expression that occur in the regenerating liver may not contribute significantly to target mRNA regulation. We also show that inclusion of HITS-CLIP data enhances miRNA target predictions in hepatocytes by more than 20-fold over computational approaches alone. These findings provide a framework for understanding the relationship of miRNA regulation to changes in gene expression and will greatly facilitate the determination of candidate ceRNA networks.

## Methods

### Animals

8–10 week old male C57Bl/6 mice underwent partial hepatectomy as described [52] using isofluorane anesthesia between 8 AM and noon, and livers were harvested at the indicated time points after partial hepatectomy. All procedures involving mice were conducted in accordance with approved Institutional Animal Care and Use Committee protocols at the University of Pennsylvania and Thomas Jefferson University.

### High-throughput sequencing of RNA isolated by cross-linking immunoprecipitation (HITS-CLIP)

At the time of tissue harvest, approximately 50% of the left lateral lobe was coarsely homogenized with a Dounce homogenizer and the resulting suspension cross-linked three times on ice in a Stratagene Crosslinker at 400 mJ/cm^2^. HITS-CLIP was performed using the monoclonal argonaute antibody 2A8 [32] as described [28]. Libraries were generated for RNA-seq and Ago HITS-CLIP (both mRNA and miRNA fraction) for quiescent liver, and liver 1 hr, 36 hr and 48 hr post partial hepatectomy, using two biological replicates for each time point. Additional information is available in Additional file 8 on-line.

### Quantification of miRNA expression

Total RNA was extracted from frozen quiescent liver and 1, 36, and 48 h post partial hepatectomy using the Qiagen miRNeasy Mini Kit (Cat. No. 217004, Valencia, CA). Real-time quantitative polymerase chain reaction (qRT-PCR) was performed as previously described [53]. 100ng of total RNA was reverse transcribed using TaqMan MicroRNA Reverse Transcription Kit (Applied Biosystems, Cat. No. 4366596, Carlsbad, CA) and RT primers from the respective TaqMan MicroRNA Assay kit (Applied Biosystems, part number 4427975 – probe numbers listed separately in Table 1). qRT-PCR was performed on a Agilent Mx3005P using the TaqMan Universal PCR Master Mix (Applied Biosystems part number 4304437) and the TaqMan probe from the respective TaqMan MicroRNA Assay kit. Tissue miRNA levels were normalized to endogenous snoRNA 202. Statistical significance of miRNA expression across various time points was calculated using One-Way ANOVA on GraphPad Prism (version 6.0). Null hypotheses were rejected at p-value *p* < 0.05. For all miRNAs, *F-*statistic was not significant.

**Table 1:** Taqman probes used for analysis of miRNA expression

**Part #**	**Assay ID**	**Description**
4427975	001200	mmu-miR-215-5p
4427975	000439	mmu-miR-103-1-3p
4427975	000464	mmu-miR-142-3p
4427975	000465	mmu-miR-142-5p
4427975	002676	mmu-miR-144-3p
4427975	002308	mmu-miR-17-5p
4427975	002493	mmu-miR-21-3p
4427975	000528	mmu-miR-301a-3p
4427975	000185	mmu-miR-31-5p
4427975	002482	mmu-miR-376a-5p
4427975	002243	mmu-miR-378-3p
4427975	001516	mmu-miR-425-5p
4427975	002509	mmu-miR-324-3p
4427975	000435	mmu-miR-99a-5p
4427975	000491	mmu-miR-192
4427975	001232	snoRNA202

### RNA-seq heat map

To construct the heat map, the log_2_ of expression levels in reads per kilobase per million (RPKM) were quantile-normalized (R normalizeBetweenArrays from the limma package) and averaged across the two replicates. mRNAs expressed at a level of at least 2 RPKM were used. K-means clustering was then performed using a range of K values from 4 to 30. K = 12 yielded a set of mostly unique profiles with a few repeated profiles indicating that K was large enough to identify the major patterns. Functional analysis of the transcripts in each cluster was performed using the ‘Core Analysis’ function at the Ingenuity website (http://www.ingenuity.com).

### Total regulatory load heat map

To construct the heat map, we calculated the total regulatory load (TRL) for all RefSeq transcripts, averaged over the two biological replicates, then followed the method used above for the RNA-Seq heat map. The strength of an individual footprint was calculated as the reads per million (RPM) in the footprint divided by the expression level of the mRNA (RPKM). The total regulatory load is the sum of the footprint strengths for all footprints on a given mRNA. We included all mRNAs with a TRL over 1 [RPM/RPKM]. A workflow diagram of computational methods is summarized in Additional file 9: Figure S3.

### Contrasting mRNA and TRL levels

We selected genes in pathways enriched in the TRL k-means clusters and plotted both mRNA and TRL profiles for these genes. The k-means clustering ensures that the TRL profiles are similar, but mRNA levels are not constrained by this process.

### miRNA-mRNA targeting prediction

Raw miRNA targeting relationships were predicted using miRanda with settings of ‘-en −10 -sc 140^′^ on all RefSeqs. A miRNA-mRNA targeting relationship was considered to be confirmed if the miRNA was present in the Ago-short library at a minimum of 100 RPM, and had a miRNA regulatory element that fell within 5 to 43 bp downstream of the start of a footprint with strength of at least 0.31 RPM/RPKM. This analysis was repeated separately for each replicate at each time point.

### Overlap with miR-29a-3p and miR-122-5p regulated genes

The set of genes, up- or down-regulated upon antisense oligonucleotide-mediated inhibition [44-46], were intersected with the set of genes that we identified as regulated by miR-29a-3p or miR-122-5p as appropriate, as well as the sets for all other miRNAs for which we could identify targets. The statistical significance of the overlaps was computed using a one-sided Fisher exact test (R function fisher.exact), then corrected for multiple testing using the Benjamini-Hochberg correction (R function p.adjust.) Additional information is available in Additional file 8 on-line.

### ceRNA networks

To identify potential ceRNA networks, we selected all mRNAs that had one major footprint targeted by a single miRNA. For this analysis, footprints within 20 bp of each other were merged. If (1) the second strongest distinct footprint was less than 25% of the strongest footprint, and (2) the most loaded miRNA in the strongest footprint was 10x higher than the second-most loaded miRNA in the footprint, then the mRNA and miRNA were included in a ceRNA network. This process was performed for each time point. Networks for each miRNA were then merged across all time-points to create summary networks.

### Availability of supporting data

All sequencing data are available through ArrayExpress. Accession number: E-MTAB-1612.

## Abbreviations

HITS-CLIP: High-throughput sequencing of RNA isolated by cross-linking immunoprecipitation; miRNAs: microRNAs; ceRNAs: Competing endogenous microRNAs; MREs: microRNA regulatory elements; TRL: Total regulatory load.

## Competing interests

The authors have declared that they have no competing interests.

## Authors’ contributions

JS carried out all bioinformatics analyses and helped to draft the manuscript. LM carried out the HITS-CLIP assays on the regenerating mouse livers. GW participated in the bioinformatics analysis. NJH and JRF participated in the design of the in silico overlap analyses with the HITS-CLIP data. SM, KE, MN, KM and YG participated in analyses of miRNA expression. VK carried out Taqman miRNA assays. AV participated in the HITS-CLIP assays and contributed to drafting of the manuscript. KHK participated in the design and coordination and helped to draft the manuscript. LEG conceived the study, performed partial hepatectomies and participated in its design and coordination and helped to draft the manuscript. All authors read and approved the final manuscript.

## Supplementary Material

Additional file 1 Schug Figure S1. Click here for file

Additional file 2: Table S1 This contains the maximum (Max), minimum (Min), and max/min fold change (FC) across the quiescent liver and post-partial hepatectomy time course, as well as the average and individual values for each time point. All data are in reads per million. Click here for file

Additional file 3 Schug Figure S2. Click here for file

Additional file 4: Table S2 This file contains the average total regulatory load (TRL) for RefSeq sequences. We show the average values at each time point as well as the minimum (Min), maximum (Max), and max/min fold change (FC). The values are calculated by adding the total RPM values for all Ago footprints on each RefSeq, then dividing by the RNA-Seq expression values for RefSeqs in reads per million per kilobase (RPKM). Click here for file

Additional file 5: Table S3 This file contains the significantly enriched Ingenuity functions in the K-means clustering of the total regulatory load (TRL) profiles. The functions are sorted by increasing p-value and by function. A function is first listed with its best Benjamini-Hochberg corrected p-value (FDR), and subsequent rows list any other clusters where the function is enriched. The genes listed are those in the cluster that are associated with the function. The p-value is corrected for multiple testing. The Cluster number corresponds to those in Figure 2A. Click here for file

Additional file 6: Table S4 This file contains the significantly enriched Ingenuity canonical pathways in the K-means clustering of the total regulatory load (TRL) profiles. The pathways are sorted by decreasing statistical significance and by name. A function is first listed with its best significance, and subsequent rows list any other clusters where the pathway is enriched. The genes listed are those in the cluster that are associated with the pathway. The FDR is the Benjamini-Hochberg corrected p-value calculated by Ingenuity. The Cluster number corresponds to those in Figure 2B. Click here for file

Additional file 7: Table S5 Positions and strengths of mRNA footprints and targeting miRNAs. Click here for file

Additional file 8 Supplemental methods. Click here for file

Additional file 9 Schug Figure S3. Click here for file
