# Supplementary figures and images for: Dynamic recruitment of microRNAs to their mRNA targets in the regenerating liver
Source: BMC Genomics. 2013 Apr 18;14:264. doi: 10.1186/1471-2164-14-264 (PMC3639193; doi:10.1186/1471-2164-14-264)

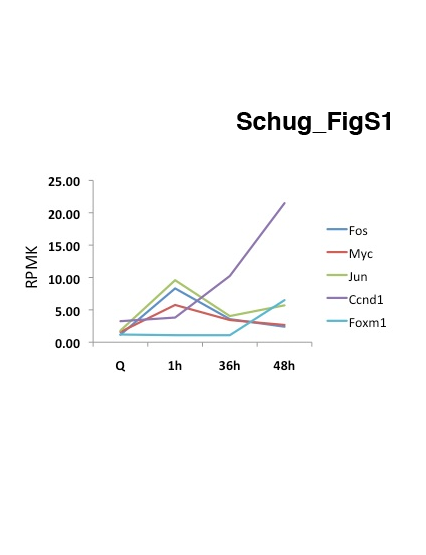

Supplement: Additional file 1 — Schug Figure S1. [file 1471-2164-14-264-S1.tiff]

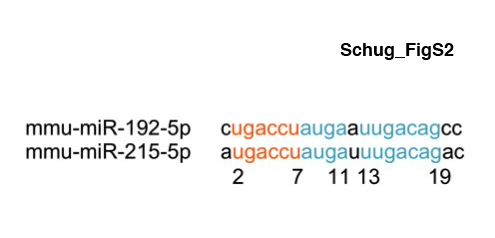

Supplement: Additional file 3 — Schug Figure S2. [file 1471-2164-14-264-S3.tiff]

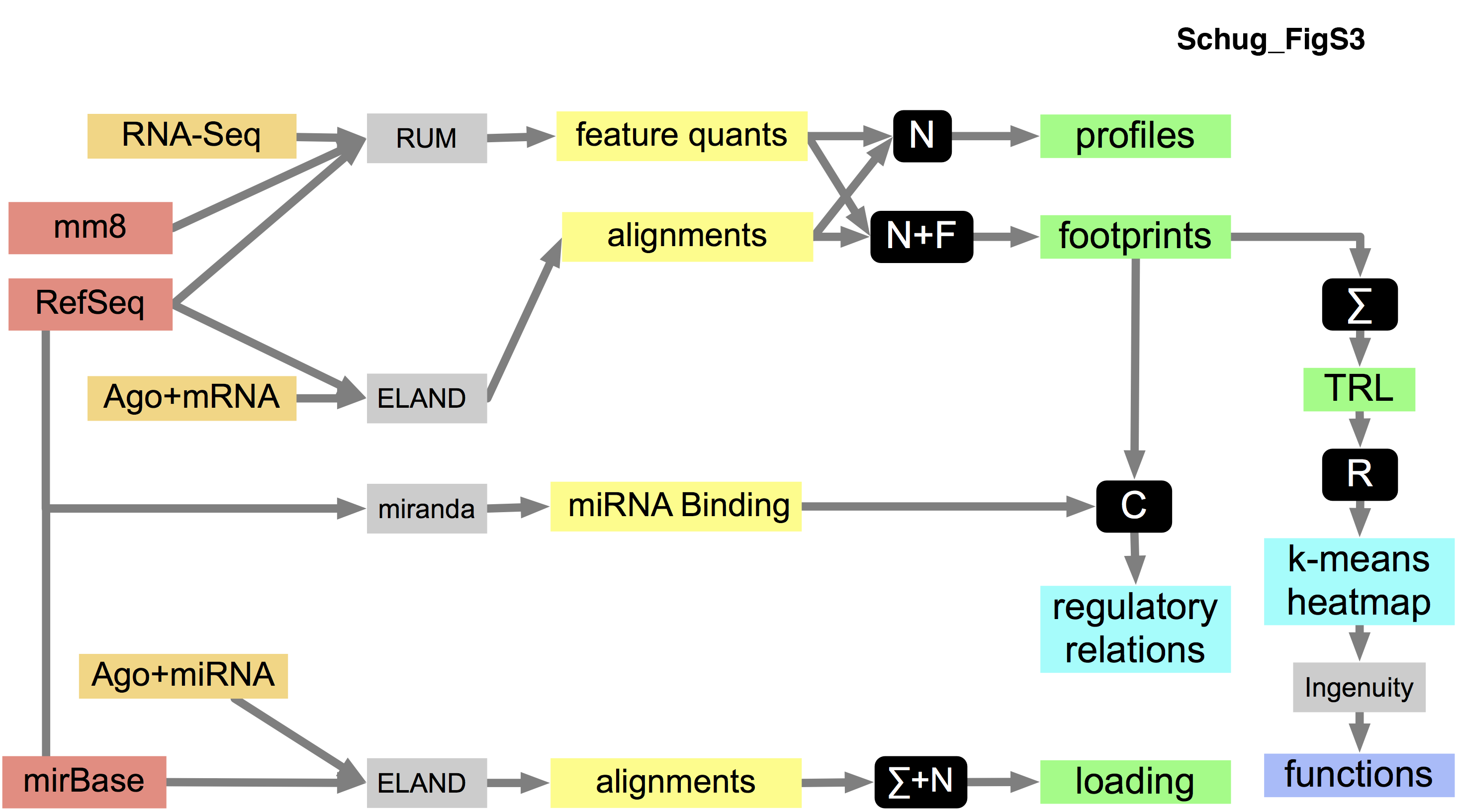

Supplement: Additional file 9 — Schug Figure S3. [file 1471-2164-14-264-S9.tiff]
