# Supplementary material for: Dynamic recruitment of microRNAs to their mRNA targets in the regenerating liver
Source: BMC Genomics. 2013 Apr 18;14:264. doi: 10.1186/1471-2164-14-264 (PMC3639193; doi:10.1186/1471-2164-14-264)
Supplement: Additional file 8 — Supplemental methods. [file 1471-2164-14-264-S8.docx]

**Supplemental methods**

**Mir-29 overlap predictions**:

We predicted 592 targets for miR-29a-3p and 1,525 targets for miR-122-5p using our combination of HITS-CLIP data and miRanda predictions. The data in Hand et al., Esau et al., and Krützfeldt et al. were split into two lists, encompassing the mRNAs that were up- or down-regulated in response to interference with miR-29a-3p and miR-122-5p activity in the liver, respectively. The overlaps are shown in the following table.

| **miRNA** | **Source** | **Gene Set** | **miRNA Targets** | **Overlap** | **Affected Genes** | **P-value** |
| --- | --- | --- | --- | --- | --- | --- |
| miR-29a-3p | Hand | Up | 592 | 76 | 22 | 2.37e-10 |
| miR-29a-3p | Hand | Down | 592 | 62 | 3 | 0.048 |
| miR-122-5p | Esau | Up | 1525 | 49 | 206 | 2.65e-06 |
| miR-122-5p | Esau | Down | 1525 | 32 | 619 | 1 |
| miR-122-5p | Krützfeldt | Up | 1525 | 93 | 363 | 3.69e-15 |
| miR-122-5p | Krützfeldt | Down | 1525 | 16 | 305 | 1 |

The statistical significance of the overlap between two data sets (predicted targets and genes with altered mRNA levels) depends on the size of the complete set of ‘all’ genes from which the two sets are drawn. The smaller the complete set, the less significant a given overlap is. Because our miRNA target sets and the 29a-3p or the miR-122-5p targets are drawn from the set of genes expressed in liver rather than all genes, we employed several different sizes for the complete set and found the overlap with the upregulated mRNAs was always significant. The number of RefSeq gene symbols was 27,350, the number of genes expressed above 1 RPKM was about 21,000, and the number of genes expressed above 3 RPKM is about 10,000. Results above are reported for RefSeq 15,000 genes.

**HITS-CLIP library preparation:**

HITS-CLIP libraries were prepared for two replicates of each condition using the protocol described in [[1](#_ENREF_1)]. Libraries were sequenced to 36bp on an Illumina GA-IIx for a minimum of 20 million reads per sample. Reads from all libraries where cleaned by trimming off trailing adapter sequences. miRNA libraries where aligned to precursor miRNA sequences using ELAND, allowing for up to two mismatches. mRNA footprints where aligned to RefSeqs using BLAT [[2](#_ENREF_2)] and allowed short insertions to accommodate the small deletions observed in mRNA footprints were created for each replicate by coalescing aligned reads into groups as follows. For each position on each RefSeq we calculated the total number of reads with an alignment that started at that position. We then arranged all locations in order from high to low, i.e., from most aligned reads to least. We processed each position in this order. A position created a new footprint if it was at least 10bp away from an existing footprint, otherwise it was added into the nearby pre-existing footprint. The final start position of a footprint was defined as the position of the first position that was used to create the footprint. The strength of a footprint is the total count of all reads that were assigned to a footprint.

Since the apparent strength of a footprint is determined both by sequencing depth and expression level of the mRNA, we normalized footprint strength to reads per HITS-CLIP million reads, then divided by the RPKM value of the RNA-Seq data to create a relative footprint strength. Further analysis of footprints was done using footprints that contained at least 10 reads. We define the *total regulatory load* (TRL) for a transcript as the sum of all the relative footprint strengths for all footprints on the transcript.

Comparison of miRNA loading levels across time was done by computing the RPM values of miRNA (using just the reads that aligned to miRNA hairpins) then performing quantile normalization. Statistical significance was assessed using ANOVA analysis followed by Tukey’s ‘honestly significant difference’ test to identify the significance of individual comparisons. ANOVA p-values were corrected for multiple testing using Benjamini-Hochberg correction. Only miRNAs with a maximum loading over 1,000 RPM were considered.

**RNAseq Data**

Libraries were constructed from 200ng of total RNA using the Illumina TruSeq mRNA V1 sample prep kit. Libraries were sequenced to 100bp (single end) on an Illumina hiSeq2000 yielding a minimum of 37 million reads for two replicates of each condition. Repeat- and ribosomal-containing reads were reduced using Bowtie [[3](#_ENREF_3)] to align the first 50bp of each read to a library of RepBase repeats and ribosomal sequences. The remaining reads where then processed with RUM [[4](#_ENREF_4)] to yield expression values for transcripts from RefSeqs, UCSC known genes, and ENSEMBL genes. Only RefSeqs were used in further analyses.

**II. Supplemental Figure Legends**

**S1:** Expression of cell cycle markers at indicated times after partial hepatectomy determined from RNAseq data. Y-Axis, RPKM: Reads per kilobase per million mapped reads.

**S2:** Alignment of mir-192-5p and mir-215-5p mature miRNA sequences. Orange nucleotides represent seed region sequence of respective miRNAs. Blue sequences represent location homologous sequences outside of seed region.

**Figure S3.** Our sequencing data (orange) was aligned (gray) to public databases  (red) to produce the primary data (yellow).  Subsequent processing  (black) created normalized profiles of RISC loading and footprints  (green) which were totaled to determined the total regulatory  load.  Total normalized miRNA content of Ago footprints and total  miRNA expression levels were also calculated (green).  Footprints and  predicted miRNA binding sites were combined to identify the miRNA:mRNA  regulatory relationships (cyan).  Total regulatory load was clustered  to produce k-means heat maps (cyan) and cluster members were processed  with Ingenuity to identify enriched functions and pathways (blue).   See Methods for details.

**References**

1. Chi SW, Zang JB, Mele A, Darnell RB: **Argonaute HITS-CLIP decodes microRNA-mRNA interaction maps**. *Nature* 2009, **460**(7254):479-486.

2. Zhang C, Darnell RB: **Mapping in vivo protein-RNA interactions at single-nucleotide resolution from HITS-CLIP data**. *Nat Biotechnol* 2011, **29**(7):607-614.

3. Langmead B, Trapnell C, Pop M, Salzberg SL: **Ultrafast and memory-efficient alignment of short DNA sequences to the human genome**. *Genome Biol* 2009, **10**(3):R25.

4. Grant GR, Farkas MH, Pizarro AD, Lahens NF, Schug J, Brunk BP, Stoeckert CJ, Hogenesch JB, Pierce EA: **Comparative analysis of RNA-Seq alignment algorithms and the RNA-Seq unified mapper (RUM)**. *Bioinformatics* 2011, **27**(18):2518-2528.
